# Supplementary material for: Transmission Pathways of the VNN Introduced in Croatian Marine Aquaculture
Source: Pathogens. 2022 Mar 30;11(4):418. doi: 10.3390/pathogens11040418 (PMC9026106; doi:10.3390/pathogens11040418)
Supplement: Supplementary file 1 [file pathogens-11-00418-s001.zip › pathogens-1636389-supplementary.pdf]

Table S1. Cummulative monthly mortalities noted for four different batches of ESB seeded in cages of the farm B and infected with NNV

| Month | Batch imported in May-14 | Batch imported in Sept-13 | Batch imported in March-14 | Batch imported in Sept-14 |
|---|---|---|---|---|
| May-14 | 678 | 447 | 187 | |
| Jun-14 | 985 | 499 | 272 | |
| Jul-14 | 4376 | 255 | 988 | |
| Aug-14 | 12403 | 483 | 1793 | |
| Sep-14 | 5724 | 1578 | 1593 | 940** |
| Oct-14 | 2999 | 542 | 716 | 344 |
| Nov-14 | 1267 | 228 | 487 | 314 |
| Dec-14 | 1496 | 257 | 254 | 179 |
| Jan-15 | 854 | 62 | 70 | 350 |
| Feb-15 | 347 | 62 | 79 | 336 |
| Mar-15 | 647 | 204 | 16 | 399 |
| Apr-15 | 591 | 59 | 5 | 523 |
| May-15 | 1207 | 101 | 41 | 447 |
| Jun-15 | 525 | 64 | 111* | 499 |
| Jul-15 | 856 | 0 | | 255 |
| Aug-15 | 426 | 0 | | 483 |

*batch imported in March 2014 was fished out in July 2015

**Batch was imported into farm

Figure S1. Heavy parasitic infection with *Diplectanum aequans* in ESB infected with NNV observed in Farm C. Note by naked eye visible abundance of parasites on the inner part of the gill arch (A) and skin of the dorsal part of the head (B).

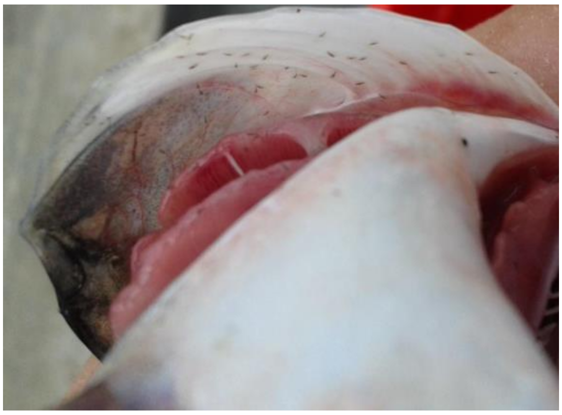

**(A)**

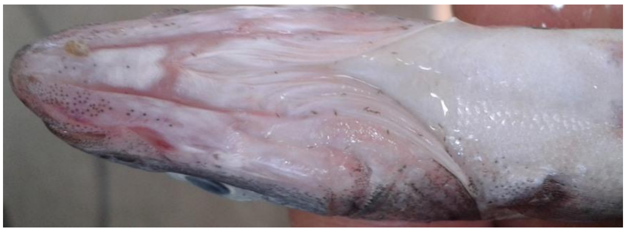

**(B)**
